# Supplementary material for: Gene Expression Profiling as a Potential Tool for Precision Oncology in Non-Small Cell Lung Cancer
Source: Cancers (Basel). 2021 Sep 22;13(19):4734. doi: 10.3390/cancers13194734 (PMC8507534; doi:10.3390/cancers13194734)
Supplement: Supplementary file 1 [file cancers-13-04734-s001.zip › cancers-1395887-SI.pdf]

cancers

MDPI

Supplementary Materials: Gene Expression Profiling as a Potential Tool for Precision Oncology in Non-Small Cell Lung Cancer

Sara Hijazo-Pechero, Ania Alay, Raúl Marín, Noelia Vilariño, Cristina Muñoz-Pinedo, Alberto Villanueva, David Santamaría, Ernest Nadal, Xavier Solé

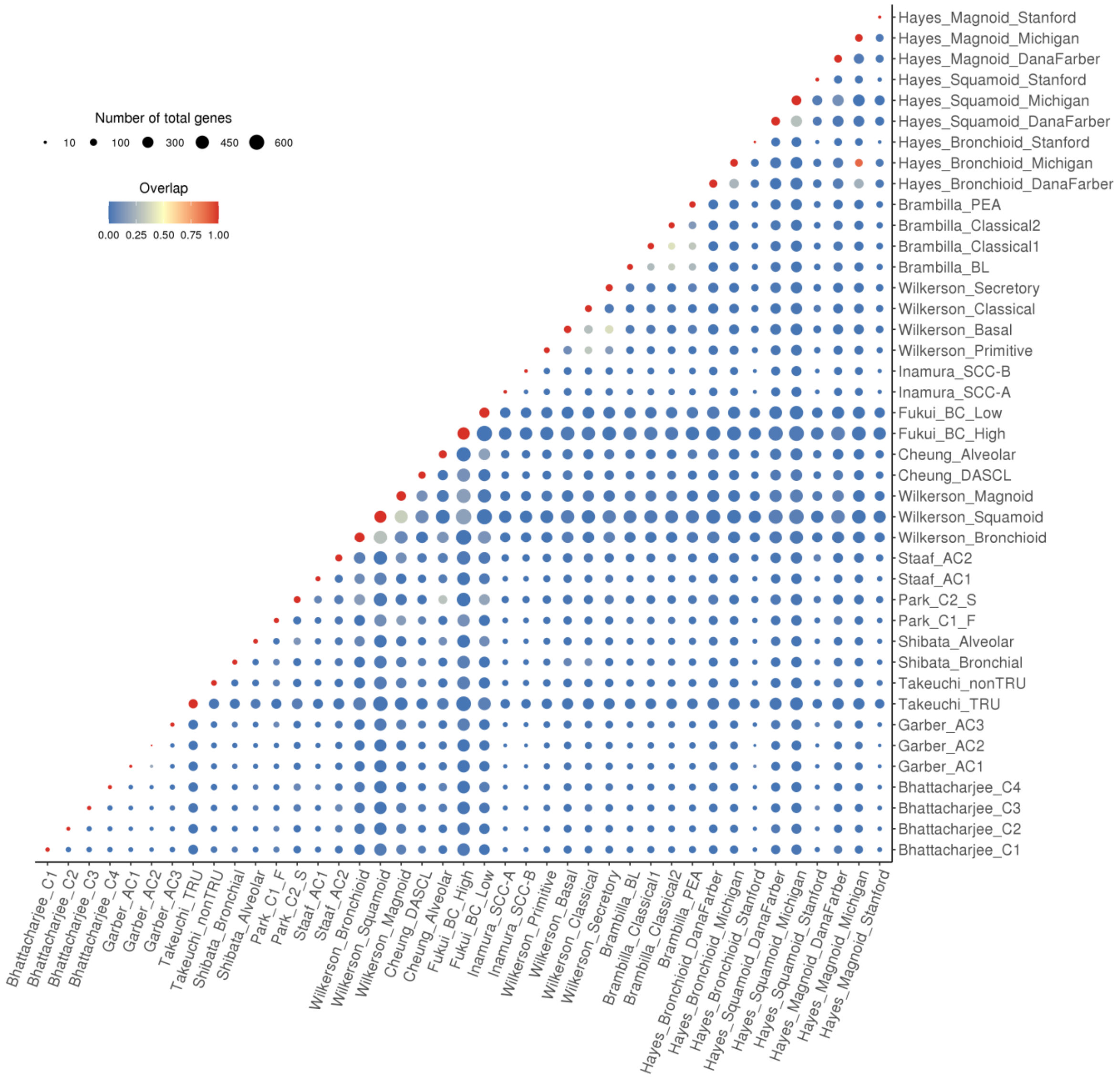

Figure S1. NSCLC transcriptional subtypes signatures overlap. This plot represents the degree of overlap between each pair of transcriptional subtypes signatures as the number of genes in common divided by the total number of genes in both signatures. Thus, gene overlap values range between 0 (blue) and 1 (red), being 1 the highest grade of overlap (all genes in common between the two signatures). Therefore, to compute the overlap we performed two previous steps detailed hereafter to construct a comparable set of signatures. **1)** Gene signature extraction: subtype specific signatures were extracted directly as gene lists when available. For the studies that provide a centroid-classifier (i.e., unique list of genes with different weights depending on the subtype), we assigned to each subtype the genes that with positive weights for that specific subtype. In the case of those studies that after the gene expression-based classification through clustering analysis performed a differential gene expression approach between the identified groups, we assigned to each subtype the genes that were overexpressed in that subtype according to the fold change provided. Finally, we excluded those studies that did not provide any further gene lists after performing the gene expression-based classifica-

tion. **2)** Gene name homogenization: the next step set out to adapt all signatures to a common identifier as they were defined by gene symbols, probe identifiers or unigene identifiers. Some signatures became shorter as some of the identifiers were deprecated and/or no associated gene symbol was found for them. The reason for this is that some of these studies are very old and used very old microarray platforms to do the transcriptional profiling. The results show that in general there is little overlap between gene expression signatures from different studies.

Table S1. Intrinsic lung adenocarcinoma transcriptional subtypes and their association with genomic alterations, molecular features and clinical covariates.

Signature	Subtype	DNA Alterations	Molecular Features	Clinical Covariates
Bhattacharjee et al. 2001 [1] 100 genes	C1	-	- Proliferation<br>- Cell division	- Poor differentiation
	C2	-	- Proliferation<br>- Neuroendocrine markers<br>- Ornithine decarboxylase 1<br>- Surfactant proteins	- Worse survival outcome
	C3	-	- Ornithine decarboxylase 1<br>Surfactant proteins<br>- Type II pneumocyte expression	- Well differentiated
	C4	-	- Type II pneumocyte expression<br>Surfactant proteins	- Well differentiated<br>- BAC* histology<br>- Stage I tumors<br>- Lower smoking history<br>- Better survival outcome
Garber et al. 2001 [2] 146 genes	AC1	-	- Surfactant proteins	- Well differentiated<br>- Good prognosis<br>- Glandular differentiation
	AC2	-	- Surfactant proteins<br>- p16 expression	- Heterogeneous differentiation<br>- Better survival outcome
	AC3	-	- p16 expression<br>- Tissue remodeling<br>- Angiogenesis<br>- *WNT* signaling inhibitor<br>- Metabolic enzymes	- Metastatic tumors<br>- Worse survival outcome<br>- Poor differentiation
Beer et al. 2002 [3] 4966 genes	Cluster 1	-		- Well differentiated<br>- BAC histology
	Cluster 2	-		
	Cluster 3	-		- Poor differentiation<br>- Stage III tumors<br>- Worse survival outcome
Tomida et al. 2004 [4] 829 genes	AD1	-	- Low peripheral lung markers expression	
	AD2	-	- High EST*** expression	
	AD3	-	- Peripheral lung markers expression	- Females<br>- Non-smokers<br>- Well differentiated
	AD4	-	- Low EST expression	
Hayes et al. 2006 [5] 2555 genes	Bronchioid	*EGFR*	- Growth, development differentiation and survival<br>- Extracellular matrix and matrix metalloproteinases<br>- Type II pneumocyte<br>- Cisplatin resistance, radiation and DNA repair	- Well differentiated<br>- Females<br>- Non-smokers<br>- BAC histology<br>- Better survival outcome
	Squamoid	*KRAS*	- WNT-HDAC2, APC (decreased)<br>- Angiogenesis	- Poor differentiation<br>- Worse survival outcome

			- Complement<br>- Zellweger's syndrome<br>- Translation<br>- Chemotherapy response	
	Magnoid		- Inflammatory genes<br>- Cytoskeleton<br>- Hematopoietic markers<br>- Lung/epithelial markers<br>- Proliferation<br>- Chemotherapy response<br>- Neuroendocrine markers	- Poor differentiation<br>- Worse survival outcome
Takeuchi et al.<br>2006 [6]<br>293 genes	TRU	*EGFR*	- Surfactant proteins<br>- Type II pneumocyte<br>- Normal lung functions	- Well differentiated<br>- Females<br>- Non-smokers<br>- Better survival outcome
	non-TRU	*KRAS, TP53*	- Proliferation<br>- Cell division<br>- Necrosis<br>- Invasive growth	- Worse survival outcome
Shibata et al.<br>2007 [7]<br>78 genes	Bronchial	*KRAS*	- Cell cycle<br>- Proliferation<br>- Extracellular matrix<br>- Bronchial epithelium	- Males<br>- Poor differentiation<br>- Solid growth<br>- Worse survival outcome
	Alveolar	*EGFR*	- Growth factor inhibitors<br>- Lipid metabolism<br>- Alveolar pneumocyte markers	- Lepidic growth<br>- Better survival outcome
Park et al.<br>2012 [8]<br>191 genes	F_C1		- Cell cycle<br>- Proliferation<br>- Loss of p53 activity	- Worse survival outcome<br>- Late stages<br>- Benefit adjuvant therapy
	S_C2			- Better survival outcome<br>- Early stages
Staaf et al.<br>2012 [9]<br>176 genes	AC1		- Increased EGFR activity	- Non-smokers
	AC2		- KIT overexpression<br>- Proliferation	- Smokers
Wilkerson et al.<br>2012 [10]<br>506 genes	Bronchioid	*EGFR*	- Excretion genes<br>- Asthma genes<br>- Surfactant proteins	- Females<br>- Non-smokers<br>- Early stage<br>- Low grade tumors<br>- Acinar content<br>- Less necrosis<br>- Less invasion<br>- Adenosquamous features<br>- Better survival outcome<br>- Potential EGFR-TKI response
	Squamoid		- Immune response genes	- High grade tumors<br>- Solid content<br>- Lowest papillary content<br>- Adenosquamous features<br>- Worse survival outcome
	Magnoid	*TP53*<br>*KRAS*<br>*STK11*	- DNA repair genes<br>- Highest mutation rate<br>- Hypermethylation	- Smokers<br>- Worse survival outcome
Cheung et al.<br>2013 [11]<br>249 genes	Alveolar		- Surfactant proteins<br>- GATA family binding sites	- Well differentiated<br>- Better survival outcome
	Distal airway stem cell-like		- WNT expression<br>- E2F and MYC expression	- Poor differentiation<br>- Epithelial features

	(DASC)			- Metastasis and progression<br>- Worse survival outcome
Fukui et al.<br>2013 [12]<br>1829 genes	BC-Low		- Type II pneumocyte expression<br>- Surfactant proteins	- Better survival outcome
	BC-High		- Cell cycle<br>- Extracellular matrix<br>- TP53 pathway<br>- Epithelial mesenchymal transition<br>- TGF-B	- Worse survival outcome
Collison et al.<br>2014 [13]<br>506 genes	TRU	*EGFR*	- Excretion genes<br>- Asthma genes<br>- Surfactant proteins	- Better survival outcome<br>- Acinar content
	PI	*TP53*<br>*NF1*	- Immune response genes	- Lepidic content<br>- Worse survival outcome
	PP	*KRAS*<br>*STK11*	- DNA repair genes<br>- Proliferation	- Worse survival outcome
Ringnér et al.<br>2016 [14]<br>Consensus classification	CONSENSUS_1	*EGFR*	- Surfactant proteins<br>- More dependent on oncogenic alterations	- Better survival<br>- Early stage<br>- Non-smokers<br>- Females
	CONSENSUS_2	*KRAS*	- Proliferation<br>- Decreased immune response<br>- Decreased extracellular matrix genes<br>- Smoking-related mutational signature	- Worse survival<br>- Younger patients<br>- Smokers
	CONSENSUS_3		- Proliferation<br>- Extracellular matrix genes<br>- Immune response<br>- Immune infiltration<br>- Basal/squamous related genes<br>- Smoking-related mutational signature	- Worse survival<br>- Lower tumor purity<br>- Smokers
Chen et al.<br>2017 [15]<br>700 genes	AD.1	*STK11*<br>*TP53*	- Cancer testis antigens expression	- LCNEC features<br>- Potential platinum-based therapy sensitivity<br>- Potential immunotherapy sensitivity
	AD.2	*KRAS*<br>*TP53*	- CpG island methylator phenotype<br>- Immune infiltration<br>- Immune checkpoints expression	- Potential immune checkpoint inhibitors therapy sensitivity
	AD.3	*TP53*	- Immune infiltration<br>- Cancer testis antigens expression<br>- Immune checkpoints expression	- Potential immunotherapy sensitivity<br>- Potential immune checkpoint inhibitors therapy sensitivity
	AD.4		- Immune infiltration<br>- Immune checkpoints expression<br>- Low neoepitope count<br>- Low mutation rate	- Better survival outcome<br>- Potential p38-related therapy sensitivity<br>- Potential mTOR-related therapy sensitivity<br>- Potential immune checkpoint inhibitors therapy sensitivity
	AD.5a		p38 pathway activationmTOR pathway activationLow mutation rate	- Non-smokers<br>- Better survival outcome<br>- Potential p38-related ther-

				apy sensitivity<br>- Potential mTOR-related therapy sensitivity
	AD5.b	*KRAS*<br>*STK11*	- CpG island methylator phenotype<br>- Low mutation rate<br>- p38 pathway activation<br>- mTOR pathway activation<br>- Low smoking-related mutational signature	- Non-smokers<br>- Better survival outcome
Hu et al.<br>2019 [16]<br>30 genes	Subtype 1	*TP53*	- Immune-related biological processes	- Worse survival outcome
	Subtype 2	*TP53*	- Immune-related biological processes	- Worse survival outcome
	Subtype 3		- Chromatid cohesion<br>- Mitotic nuclear division<br>- Cell division	
	Subtype 4	*EGFR*	- Collagen fibril organization<br>- Collagen catabolic process<br>- Extracellular matrix organization<br>- PI3K-Akt signaling<br>- Focal adhesion	- Better survival outcome

Table S2. Intrinsic lung squamous cell carcinoma transcriptional subtypes and their association with genomic alterations, molecular features and clinical covariates.

Signature	Subtype	DNA Alterations	Molecular Features	Clinical Covariates
Tomida et al.<br>2004 [4]<br>829 genes	SQ1	-	- Extracellular matrix<br>- Proliferation<br>- Infiltrative growth pattern<br>- High keratinization	- Worse survival outcome
	SQ2	-	- Well circumscribed expansive growth pattern<br>- Low keratinization	- Better survival outcome
Inamura et al.<br>2005 [17]<br>3344 genes	SCC-A	-	- Proliferation	- Worse survival outcome
	SCC-B	-	- *AKT2* overexpression<br>- MAPKK cascade<br>- Ceramide metabolism<br>- Apoptosis	- Better survival outcome
Raponi et al.<br>2006 [18]<br>11101 genes	Cluster 1	-		- Worse survival outcome
	Cluster 2	-		- Better survival outcome
Larsen et al.<br>2007 [19]<br>6748 genes	Cluster 1	-		- Worse survival outcome<br>- Poor differentiation
	Cluster 2	-		- Better survival outcome
Wilkerson et al.<br>2010 [20]<br>208 genes	Primitive	-	- Proliferation<br>- DNA repair	- Poor differentiation<br>- Females<br>- Worse survival outcome
	Basal	-	- *S100A8* expression<br>- Cell adhesion<br>- Epidermal development	- Well differentiated
	Secretory	-	- *KRT7* expression<br>- Immune response<br>- T cell activation<br>- Positive regulation NFKb pathway<br>- Surfactant proteins	- Stage III tumors
	Classical	3q27-28 amplification	- *TP63* expression<br>- Oxidative metabolism<br>- Xenobiotic metabolism	- Smokers<br>- Stage III tumors<br>- Males
Hammerman et al.<br>2012 [21]	Primitive	*RB1*<br>*PTEN*	- Proliferation<br>- DNA repair	

208 genes	Basal	*NF1*	- *S100A8* expression<br>- Cell adhesion<br>- Epidermal development	
	Secretory		- *KRT7* expression<br>- Immune response<br>- T cell activation<br>- Positive regulation NFKb pathway<br>- Surfactant proteins	
	Classical	*KEAP1*<br>*NFE2L2*<br>*PTEN*<br>3q26 amplification	- Hypermethylation<br>- Chromosomal instability<br>- Oxidative metabolism<br>- *SOX2* expression<br>- *TP63* expression<br>- *PI3KCA* expression	
Brambilla et al. 2014 [22] 139 genes	Classical_1	*TP53*	- Glycolysis<br>- TP53 targets<br>- Senescence<br>- Cell adhesion<br>- Apoptosis<br>- Keratins<br>- Drug metabolism	- Well differentiated<br>- Better survival outcome
	Classical_2		- Glycolysis<br>- Oxidative phosphorylation<br>- Keratins<br>- Drug metabolism<br>- Metastasis<br>- Proliferation	- Well differentiated
	PEA		- NRAS signaling<br>- Immune response<br>- Surfactant proteins<br>- Senescence<br>- TP53 targets<br>- Cell adhesion<br>- Apoptosis<br>- Cell motility	- Stage I tumors<br>- Alveolar features<br>- Poor differentiation
	BL		- Basal cells signatures expression<br>- Oxidative phosphorylation<br>- Proliferation<br>- Testis antigens expression<br>- Metastasis	- Basaloid histology<br>- Late-stage tumors<br>- Worse survival outcome
Chen et al. 2017 [15] 700 genes	SQ.1		- Epithelial mesenchymal transition<br>- *SOX2* expression<br>- *TP63* expression<br>- Immune infiltration	Worse survival outcome
	SQ.2a	*SOX2* copy gain<br>*TP53*	- *SOX2* expression<br>- Immune infiltration	Better survival outcome
	SQ.2b	*SOX2* copy gain<br>*TP53*	- *SOX2* expression	Better survival outcome

Table S3. Identified NSCLC prognostic signatures published in the last 5 years (2016-2021).

Scheme .	Year	Journal	Reference	Tissue of origin	Number of genes	Technology	Public datasets accession numbers
Chen et al.	2016	Oncotarget	[23]	AD	72	Microarray	GSE31210, GSE19188, GSE19804
Shahid et al.	2016	Oncotarget	[24]	NSCLC	8	Microarray	GSE50081, GSE31210, GSE30219, GSE29013, E-MTAB-923, GSE68465, GSE42127
Xu et al.	2016	J. Thorac. Oncol.	[25]	NSCLC	10	Microarray/RNA-Seq	GSE3141, GSE83227, GSE68465, TCGA-LUAD, GSE31210, GSE13213, GSE42127, E-MTAB-923, GSE14814, GSE11969, GSE41271, GSE50081
Krzystanek et al.	2016	Biomarker Research	[26]	AD	7	Microarray/RNA-Seq	GSE8894, GSE14814, GSE30219, GSE31210, GSE37745, GSE50081, GSE68465, TCGA-LUAD
Zhang et al.	2016	Nat. Commun.	[27]	Lung and Others	14	Microarray	GSE37745, GSE42127, GSE31210
Shukla et al.	2016	J. Natl. Cancer Inst.	[28]	AD	4	RNA-Seq	TCGA-LUAD
Li et al.	2017	JAMA Oncology	[29]	Non-SCC NSCLC	40	Micro-array/RNA-Seq	TCGA-LUAD, GSE68465, GSE30219, GSE31210, GSE41271, GSE50081, GSE13213, GSE26939, GSE83227, GSE11969, GSE42127, GSE68571, GSE19188, GSE3141, GSE37745, GSE10245, GSE14814, GSE31547, GSE31546
Namani et al.	2017	Oncotarget	[30]	NSCLC	12	Microarray/RNA-Seq	TCGA-LUAD, GSE3141, GSE8894, GSE13213, GSE31210, GSE42127, GSE37745, Roepman
Chen et al.	2017	Oncotarget	[31]	AD	27	Microarray	GSE31210, GSE19188, GSE19804
Li et al.	2017	J. Cell Physiol.	[32]	SCC	4	RNA-Seq	TCGA-LUSC
Higgs et al.	2018	Clin. Cancer Res.	[33]	NSCLC	4	RNA-Seq	1108/NCT01693562, TCGA-LUAD, TCGA-LUSC
Zheng et al.	2018	Int. J. Cancer	[34]	AD	8	Microarray/RNA-Seq	TCGA-LUAD, GSE31210, GSE50081, GSE81089, FUSCC cohort
Zhang et al.	2018	Int. J. Med. Sci.	[35]	AD	3	Microarray/RNA-Seq	GSE31210, GSE32863, GSE40791, GSE43458, GSE75037, TCGA-LUAD
Li et al.	2018	Cancer Sci.	[36]	AD, SCC	17	Microarray	GSE13213, GSE68465, GSE83227, GSE8894, GSE19188, GSE8894, GSE4573, GSE14814, GSE43767
Zuo et al.	2019	J. Transl. Med.	[37]	NSCLC	6	Microarray/RNA-Seq	GSE31210, GSE50081, GSE37745, TCGA-LUSC, TCGA-LUAD
Xie et al.	2019	Biomed. Res. Int.	[38]	NSCLC	6	Microarray/RNA-Seq	GSE19188, GSE30219, GSE10072, GSE7670, GSE2514, GSE32863, GSE21933, GSE40275, GSE12472, GSE80796, GSE8500, GSE85841, GSE19027, GSE11969, TCGA-LUAD
Wu et al.	2019	Cancer Med.	[39]	AD	5	Microarray/RNA-Seq	GSE68465, GSE13213, TCGA-LUAD
Jan et al.	2019	Sci. Rep.	[40]	AD	118	RNA-Seq	TCGA-LUAD

Wang et al.	2019	Onco. Targets Ther.	[41]	SCC	7	RNA-Seq	TCGA-LUSC
Zhang et al.	2019	J. Gene Med.	[42]	SCC	17	Microarray/RNA-Seq	TCGA-LUSC, GSE14814, GSE50081, GSE30219, GSE37745, GSE29013, GSE3141, GSE19188
Liu et al.	2019	Cell Cycle	[43]	AD	4	RNA-Seq	TCGA-LUAD
Li et al.	2020	Biomed. Res. Int.	[44]	NSCLC	5	Microarray/RNA-Seq	GSE103584, GSE31210, GSE41271, TCGA-LUAD
Liu et al.	2020	J. Cell Physiol.	[45]	NSCLC	8	RNA-Seq	TCGA-LUAD, TCGA-LUSC
Liu et al.	2020	Aging	[46]	NSCLC	7	Microarray/RNA-Seq	TCGA-LUAD, TCGA-LUSC, GSE30219, GSE41271, GSE42127, GSE50081
Li et al.	2020	Int. J. Mol. Sci.	[47]	AD	8	Microarray/RNA-Seq	GSE18842, GSE75037, GSE101929, GSE19188, TCGA-LUAD
Liu et al.	2020	Aging	[48]	NSCLC	22	Microarray/RNA-Seq	TCGA-LUSC, TCGA-LUAD, GSE31210, GSE30219, GSE3141, GSE8894
Li et al.	2020	Lipids Health Dis.	[49]	AD	6	Microarray/RNA-Seq	TCGA-LUAD, GSE13213
Li et al.	2020	Oncol. Rep.	[50]	NSCLC	10	Microarray/RNA-Seq	GSE103584, GSE31210, TCGA-LUAD, TCGA-LUSC
Fan et al.	2020	Aging	[51]	AD	4	RNA-Seq	TCGA-LUAD
Ma et al.	2020	Front. Mol. Biosci.	[52]	AD	8	Microarray/RNA-Seq	TCGA-LUAD, GSE72094
Han et al.	2020	Cancer Med.	[53]	AD	6	Microarray/RNA-Seq	GSE37745, GSE30219, TCGA-LUAD
Ma et al.	2020	Biomed. Res. Int.	[54]	SCC	5	Microarray/RNA-Seq	TCGA-LUSC, GSE42127
Bao et al.	2020	J. Mol. Med, (Berl.)	[55]	AD	12	Microarray/RNA-Seq	GSE42127, GSE37745, GSE50081, GSE29013, GSE72094, TCGA-LUAD
Yu et al.	2020	J. Oncol.	[56]	AD	5	Microarray/RNA-Seq	GSE72094, TCGA-LUAD
Tang et al.	2020	Aging	[57]	AD	9	Microarray/RNA-Seq	GSE31210, GSE41271, TCGA-LUAD
Jiang et al.	2020	BMC Cancer	[58]	AD	10	Microarray/RNA-Seq	GSE75037, TCGA-LUAD
He et al.	2020	Mol. Ther. Oncolytics	[59]	AD	13	Microarray/RNA-Seq	GSE31210, GSE30219, TCGA-LUAD
Song et al.	2020	Front. Oncol.	[60]	AD	4	Microarray/RNA-Seq	GSE68465, TCGA-LUAD
Ma et al.	2020	PeerJ.	[61]	SCC	7	Microarray/RNA-Seq	GSE19188, GSE33532, GSE33479, TCGA-LUSC
Zhao et al.	2020	PeerJ.	[62]	AD	23	Microarray/RNA-Seq	GSE30219, GSE72094, TCGA-LUAD
Sun et al.	2020	Ther. Adv. Med. Oncol.	[63]	AD	16	Microarray/RNA-Seq	GSE72094,GSE31210, GSE30219, GSE37745, GSE50081, GSE42127, GSE42127, TCGA-LUAD
Wu et al.	2020	J. Int. Med. Res.	[64]	NSCLC	5	Microarray/RNA-Seq	GSE30219, GSE37745, GSE50081, TCGA-LUAD, TCGA-LUSC
Li et al.	2020	Front. Genet.	[65]	SCC	9	Microarray/RNA-Seq	GSE73403, TCGA-LUSC
Guo et al.	2020	J. Transl. Med.	[66]	AD	10	Microarray/RNA-Seq	TCGA-LUAD, GSE31210, GSE72094
Zhang et al.	2020	Biomed. Res. Int.	[67]	AD	7	Microarray/RNA-Seq	GSE26939, TCGA-LUAD
Nema et al.	2020	Comput. Biol.Med.	[68]	AD	3	Microarray/RNA-Seq	GSE14814, GE19188, GSE29013, GSE30219, GSE31210, GSE3141, GSE31908, GSE37745, GSE37745, GSE43580, GSE4573, GSE50081, GSE8894, TCGA-LUAD

Yang et al.	2020	PeerJ.	[69]	AD	6	Microarray/RNA-Seq	GSE31210, GSE37745, TCGA-LUAD
Wu et al.	2020	J. Transl. Med.	[70]	AD	21	Microarray/RNA-Seq	GSE30219, GSE31210, GSE50081, GSE72094, TCGA-LUAD
Wu et al.	2020	Int. Immunopharmacol.	[71]	AD	7	Microarray/RNA-Seq	GSE10072, GSE31210, GSE40791, TCGA-LUAD
Zou et al.	2020	Med. Sci. Monit.	[72]	AD	7	Microarray/RNA-Seq	GSE30219, GSE41271, GSE42127, TCGA-LUAD
Li et al.	2020	BMC Cancer	[73]	AD	4	Microarray/RNA-Seq	GSE42127, TCGA-LUAD
Zengin et al.	2020	BMC Bioinformatics	[74]	AD	12	RNA-Seq	TCGA-LUAD
Zhao et al.	2020	Immunogenetics	[75]	AD	8	Microarray/RNA-Seq	GSE30219, GSE31210, GSE3141, GSE37745, GSE50081, TCGA-LUAD
Cao et al.	2020	PeerJ.	[76]	AD	6	Microarray/RNA-Seq	TCGA-LUAD, GSE68465
Zhao et al.	2020	Lung Cancer	[77]	AD	19	Microarray/RNA-Seq	GSE30219, GSE31210, GSE50081, TCGA-LUAD
Zheng et al.	2020	Biomed. Res. Int.	[78]	AD	5	Microarray/RNA-Seq	GSE30219, GSE31210, GSE50081, TCGA-LUAD
Ma et al.	2021	Sci. Rep.	[79]	NSCLC	15	Microarray	GSE30219, GSE31210, GSE50081
Yan et al.	2021	Sci. Rep.	[80]	SCC	3	Microarray/RNA-Seq	GSE73403, TCGA-LUSC
Ma et al.	2021	Aging	[81]	AD	8	Microarray/RNA-Seq	GSE31210, GSE30219, GSE13213, GSE50081, TCGA-LUAD
Zhang et al.	2021	Biomed. Res. Int.	[82]	NSCLC	6	Microarray/RNA-Seq	GSE41271, GSE81089, TCGA-LUSC, TCGA-LUAD
Yao et al.	2021	J. Cancer	[83]	NSCLC	5	RNA-Seq	TCGA-LUAD, TCGA-LUSC
Yang et al.	2021	PeerJ.	[84]	AD	11	Microarray/RNA-Seq	GSE68465, TCGA-LUAD
Ma et al.	2021	Transl. Lung Cancer Res.	[85]	AD	3	Microarray/RNA-Seq	GSE68571, GSE72094, TCGA-LUAD
Wang et al.	2021	PeerJ.	[86]	AD	5	Microarray/RNA-Seq	GSE68465, GSE41271, TCGA-LUAD
Xu et al.	2021	Cancer Med.	[87]	SCC	5	RNA-Seq	TCGA-LUSC
Tu et al.	2021	PeerJ.	[88]	AD	8	Microarray/RNA-Seq	GSE31210, GSE32665, GSE32863, GSE43458, GSE72094, TCGA-LUAD
Zhu et al.	2021	Thorac. Cancer	[89]	AD	10	Microarray/RNA-Seq	TCGA-LUAD, GSE31210
Xu et al.	2021	Aging	[90]	AD	5	RNA-Seq	TCGA-LUAD
Chen et al.	2021	PeerJ.	[91]	AD	106	Microarray/RNA-Seq	TCGA-LUAD, GSE68465, GSE14814
Yang et al.	2021	Ann. Transl. Med.	[92]	AD	9	Microarray/RNA-Seq	TCGA-LUAD, GSE13213
Miao et al.	2021	Front. Oncol.	[93]	Lung cancer coexisting with COPD	5	RNA-Seq	TCGA-LUAD, TCGA-LUSC
Wang et al.	2021	Cancer Cell Int.	[94]	AD	6	Microarray/RNA-Seq	TCGA-LUAD, GSE31210

References.

1. Bhattacharjee, A.; Richards, W.G.; Staunton, J.; Li, C.; Monti, S.; Vasa, P.; Ladd, C.; Beheshti, J.; Bueno, R.; Gillette, M.; et al. Classification of human lung carcinomas by mRNA expression profiling reveals distinct adenocarcinoma subclasses. *Proc. Natl. Acad. Sci.* **2001**, *98*, 13790–13795, https://doi.org/10.1073/pnas.191502998.
2. Garber, M.E.; Troyanskaya, O.G.; Schluens, K.; Petersen, S.; Thaesler, Z.; Pacyna-Gengelbach, M.; van de Rijn, M.; Rosen, G.D.; Perou, C.; Whyte, R.I.; et al. Diversity of gene expression in adenocarcinoma of the lung. *Proc. Natl. Acad. Sci.* **2001**, *98*, 13784–13789, https://doi.org/10.1073/pnas.241500798.
3. Beer, D.G.; Kardia, S.L.; Huang, C.-C.; Giordano, T.; Levin, A.M.; Misek, D.E.; Lin, L.; Chen, G.; Gharib, T.G.; Thomas, D.G.; et al. Gene-expression profiles predict survival of patients with lung adenocarcinoma. *Nat. Med.* **2002**, *8*, 816–824, https://doi.org/10.1038/nm733 nm733 [pii].
4. Tomida, S.; Koshikawa, K.; Yatabe, Y.; Harano, T.; Ogura, N.; Mitsudomi, T.; Some, M.; Yanagisawa, K.; Takahashi, T.; Osada, H.; et al. Gene expression-based, individualized outcome prediction for surgically treated lung cancer patients. *Oncogene* **2004**, *23*, 5360–5370, https://doi.org/10.1038/sj.onc.1207697.
5. Hayes, D.N.; Monti, S.; Parmigiani, G.; Gilks, C.B.; Naoki, K.; Bhattacharjee, A.; Socinski, M.A.; Perou, C.; Meyerson, M. Gene Expression Profiling Reveals Reproducible Human Lung Adenocarcinoma Subtypes in Multiple Independent Patient Cohorts. *J. Clin. Oncol.* **2006**, *24*, 5079–5090, https://doi.org/10.1200/jco.2005.05.1748.
6. Takeuchi, T.; Tomida, S.; Yatabe, Y.; Kosaka, T.; Osada, H.; Yanagisawa, K.; Mitsudomi, T.; Takahashi, T. Expression Profile–Defined Classification of Lung Adenocarcinoma Shows Close Relationship With Underlying Major Genetic Changes and Clinicopathologic Behaviors. *J. Clin. Oncol.* **2006**, *24*, 1679–1688, https://doi.org/10.1200/jco.2005.03.8224.
7. Shibata, T.; Hanada, S.; Kokubu, A.; Matsuno, Y.; Asamura, H.; Ohta, T.; Sakamoto, M.; Hirohashi, S. Gene expression profiling of epidermal growth factor receptor/KRAS pathway activation in lung adenocarcinoma. *Cancer Sci.* **2007**, *98*, 985–991, https://doi.org/10.1111/j.1349-7006.2007.00483.x.
8. Park, Y.-Y.; Park, E.S.; Kim, S.B.; Kim, S.C.; Sohn, B.H.; Chu, I.-S.; Jeong, W.; Mills, G.B.; Byers, L.A.; Lee, J.-S. Development and Validation of a Prognostic Gene-Expression Signature for Lung Adenocarcinoma. *PLOS ONE* **2012**, *7*, e44225, https://doi.org/10.1371/journal.pone.0044225.
9. Staaf, J.; Jönsson, G.; Jönsson, M.; Karlsson, A.; Isaksson, S.; Salomonsson, A.; Pettersson, H.M.; Soller, M.; Ewers, S.-B.; Johansson, L.; et al. Relation between smoking history and gene expression profiles in lung adenocarcinomas. *BMC Med Genom.* **2012**, *5*, 22–22, https://doi.org/10.1186/1755-8794-5-22.
10. Wilkerson, M.D.; Yin, X.; Walter, V.; Zhao, N.; Cabanski, C.R.; Hayward, M.C.; Miller, C.; Socinski, M.A.; Parsons, A.M.; Thorne, L.B.; et al. Differential Pathogenesis of Lung Adenocarcinoma Subtypes Involving Sequence Mutations, Copy Number, Chromosomal Instability, and Methylation. *PLOS ONE* **2012**, *7*, e36530, https://doi.org/10.1371/journal.pone.0036530.
11. Cheung, W.K.; Zhao, M.; Liu, Z.; Stevens, L.E.; Cao, P.D.; Fang, J.E.; Westbrook, T.F.; Nguyen, D.X. Control of Alveolar Differentiation by the Lineage Transcription Factors GATA6 and HOPX Inhibits Lung Adenocarcinoma Metastasis. *Cancer Cell* **2013**, *23*, 725–738, https://doi.org/10.1016/j.ccr.2013.04.009.
12. Fukui, T.; Shaykhiev, R.; Agosto-Perez, F.; Mezey, J.G.; Downey, R.J.; Travis, W.D.; Crystal, R.G. Lung adenocarcinoma subtypes based on expression of human airway basal cell genes. *Eur. Respir. J.* **2013**, *42*, 1332–1344, https://doi.org/10.1183/09031936.00144012.
13. The Cancer Genome Atlas Research Network Comprehensive molecular profiling of lung adenocarcinoma. *Nat. Cell Biol.* **2014**, *511*, 543–550, https://doi.org/10.1038/nature13385.
14. Ringner, M.; Staaf, J. Consensus of gene expression phenotypes and prognostic risk predictors in primary lung adenocarcinoma. *Oncotarget* **2016**, *7*, 52957–52973, https://doi.org/10.18632/oncotarget.10641.
15. Chen, F.; Zhang, Y.; Parra, E.; Rodriguez, J.; Behrens, C.; Akbani, R.; Lu, Y.; Kurie, J.M.; Gibbons, D.L.; Mills, G.; et al. Multiplatform-based molecular subtypes of non-small-cell lung cancer. *Oncogene* **2016**, *36*, 1384–1393, https://doi.org/10.1038/onc.2016.303.
16. Hu, F.; Zhou, Y.; Wang, Q.; Yang, Z.; Shi, Y.; Chi, Q. Gene expression classification of lung adenocarcinoma into molecular subtypes. *IEEE/ACM Trans. Comput. Biol. Bioinform.* **2019**, 1–1, https://doi.org/10.1109/tcbb.2019.2905553.
17. Inamura, K.; Fujiwara, T.; Hoshida, Y.; Isagawa, T.; Jones, M.; Virtanen, C.; Shimane, M.; Satoh, Y.; Okumura, S.; Nakagawa, K.; et al. Two subclasses of lung squamous cell carcinoma with different gene expression profiles and prognosis identified by hierarchical clustering and non-negative matrix factorization. *Oncogene* **2005**, *24*, 7105–7113, https://doi.org/10.1038/sj.onc.1208858.
18. Raponi, M.; Zhang, Y.; Yu, J.; Chen, G.; Lee, G.; Taylor, J.M.; Macdonald, J.; Thomas, D.; Moskaluk, C.; Wang, Y.; et al. Gene Expression Signatures for Predicting Prognosis of Squamous Cell and Adenocarcinomas of the Lung. *Cancer Res.* **2006**, *66*, 7466–7472, https://doi.org/10.1158/0008-5472.can-06-1191.
19. Larsen, J.E.; Pavey, S.J.; Passmore, L.H.; Bowman, R.; Clarke, B.E.; Hayward, N.K.; Fong, K.M. Expression profiling defines a recurrence signature in lung squamous cell carcinoma. *Carcinogenesis* **2006**, *28*, 760–766, https://doi.org/10.1093/carcin/bgl207.
20. Wilkerson, M.D.; Yin, X.; Hoadley, K.; Liu, Y.; Hayward, M.C.; Cabanski, C.R.; Muldrew, K.; Miller, C.; Randell, S.H.; Socinski, M.A.; et al. Lung Squamous Cell Carcinoma mRNA Expression Subtypes Are Reproducible, Clinically Important, and Correspond to Normal Cell Types. *Clin. Cancer Res.* **2010**, *16*, 4864–4875, https://doi.org/10.1158/1078-0432.ccr-10-0199.
21. The Cancer Genome Atlas Research Network Comprehensive genomic characterization of squamous cell lung cancers. *Nature* **2012**, *489*, 519–525, https://doi.org/10.1038/nature11404.

22. Brambilla, C.; Laffaire, J.; Lantuejoul, S.; Moro-Sibilot, D.; Mignotte, H.; Arbib, F.; Toffart, A.-C.; Petel, F.; Hainaut, P.; Rousseaux, S.; et al. Lung Squamous Cell Carcinomas with Basaloid Histology Represent a Specific Molecular Entity. *Clin. Cancer Res.* **2014**, *20*, 5777–5786, https://doi.org/10.1158/1078-0432.ccr-14-0459.
23. Chen, E.-G.; Wang, P.; Lou, H.; Wang, Y.; Yan, H.; Bi, L.; Liu, L.; Li, B.; Snijders, A.M.; Mao, J.-H.; et al. A robust gene expression-based prognostic risk score predicts overall survival of lung adenocarcinoma patients. *Oncotarget* **2017**, *9*, 6862–6871, https://doi.org/10.18632/oncotarget.23490.
24. Shahid, M.; Choi, T.G.; Nguyen, M.N.; Matondo, A.; Jo, Y.H.; Yoo, J.Y.; Nguyen, N.N.Y.; Yun, H.R.; Kim, J.; Akter, S.; et al. An 8-gene signature for prediction of prognosis and chemoresponse in non-small cell lung cancer. *Oncotarget* **2016**, *7*, 86561–86572, https://doi.org/10.18632/oncotarget.13357.
25. Xu, W.; Jia, G.; Davie, J.; Murphy, L.; Kratzke, R.; Banerji, S. A 10-Gene Yin Yang Expression Ratio Signature for Stage IA and IB Non–Small Cell Lung Cancer. *J. Thorac. Oncol.* **2016**, *11*, 2150–2160, https://doi.org/10.1016/j.jtho.2016.07.023.
26. Krzystanek, M.; Moldvay, J.; Szüts, D.; Szallasi, Z.; Eklund, A.C. A robust prognostic gene expression signature for early stage lung adenocarcinoma. *Biomark. Res.* **2016**, *4*, 1–7, https://doi.org/10.1186/s40364-016-0058-3.
27. Zhang, W.; Mao, J.-H.; Zhu, W.; Jain, A.K.; Liu, K.; Brown, J.B.; Karpen, G.H. Centromere and kinetochore gene misexpression predicts cancer patient survival and response to radiotherapy and chemotherapy. *Nat. Commun.* **2016**, *7*, 12619–12619, https://doi.org/10.1038/ncomms12619.
28. Shukla, S.; Evans, J.R.; Malik, R.; Feng, F.Y.; Dhanasekaran, S.M.; Cao, X.; Chen, G.; Etherton-Beer, C.; Jiang, H.; Chinnaiyan, A.M. Development of a RNA-Seq Based Prognostic Signature in Lung Adenocarcinoma. *J. Natl. Cancer Inst.* **2016**, *109*, https://doi.org/10.1093/jnci/djw200.
29. Li, B.; Cui, Y.; Diehn, M.; Li, R. Development and Validation of an Individualized Immune Prognostic Signature in Early-Stage Nonsquamous Non–Small Cell Lung Cancer. *JAMA Oncol.* **2017**, *3*, 1529–1537, https://doi.org/10.1001/jamaoncol.2017.1609.
30. Namani, A.; Cui, Q.Q.; Wu, Y.; Wang, H.; Wang, X.J.; Tang, X. NRF2-regulated metabolic gene signature as a prognostic biomarker in non-small cell lung cancer. *Oncotarget* **2017**, *8*, 69847–69862, https://doi.org/10.18632/oncotarget.19349.
31. Chen, E.-G.; Wang, P.; Lou, H.; Wang, Y.; Yan, H.; Bi, L.; Liu, L.; Li, B.; Snijders, A.M.; Mao, J.-H.; et al. A robust gene expression-based prognostic risk score predicts overall survival of lung adenocarcinoma patients. *Oncotarget* **2017**, *9*, 6862–6871, https://doi.org/10.18632/oncotarget.23490.
32. Li, J.; Wang, J.; Chen, Y.; Yang, L.; Chen, S. A prognostic 4-gene expression signature for squamous cell lung carcinoma. *J. Cell. Physiol.* **2017**, *232*, 3702–3713, https://doi.org/10.1002/jcp.25846.
33. Higgs, B.W.; Morehouse, C.A.; Streicher, K.L.; Brohawn, P.Z.; Pilataxi, F.; Gupta, A.; Ranade, K. Interferon Gamma Messenger RNA Signature in Tumor Biopsies Predicts Outcomes in Patients with Non–Small Cell Lung Carcinoma or Urothelial Cancer Treated with Durvalumab. *Clin. Cancer Res.* **2018**, *24*, 3857–3866, https://doi.org/10.1158/1078-0432.ccr-17-3451.
34. Zheng, S.; Luo, X.; Dong, C.; Zheng, D.; Xie, J.; Zhuge, L.; Sun, Y.; Chen, H. A B7-CD28 family based signature demonstrates significantly different prognoses and tumor immune landscapes in lung adenocarcinoma. *Int. J. Cancer* **2018**, *143*, 2592–2601, https://doi.org/10.1002/ijc.31764.
35. Zhang, M.-Y.; Liu, X.-X.; Li, H.; Li, R.; Qu, Y.-Q. Elevated mRNA Levels of AURKA, CDC20 and TPX2 are associated with poor prognosis of smoking related lung adenocarcinoma using bioinformatics analysis. *Int. J. Med Sci.* **2018**, *15*, 1676–1685, https://doi.org/10.7150/ijms.28728.
36. Li, P.; Zhang, L.; Yu, X.; Tong, R.; Di, X.; Mao, Y.; Gao, Y.; Zhang, K.; Feng, L.; Cheng, S. Proliferation genes in lung development associated with the prognosis of lung adenocarcinoma but not squamous cell carcinoma. *Cancer Sci.* **2017**, *109*, 308–316, https://doi.org/10.1111/cas.13456.
37. Zuo, S.; Wei, M.; Zhang, H.; Chen, A.; Wu, J.; Wei, J.; Dong, J. A robust six-gene prognostic signature for prediction of both disease-free and overall survival in non-small cell lung cancer. *J. Transl. Med.* **2019**, *17*, 152, https://doi.org/10.1186/s12967-019-1899-y.
38. Xie, H.; Xie, C. A Six-Gene Signature Predicts Survival of Adenocarcinoma Type of Non-Small-Cell Lung Cancer Patients: A Comprehensive Study Based on Integrated Analysis and Weighted Gene Coexpression Network. *BioMed Res. Int.* **2019**, *2019*, 1–16, https://doi.org/10.1155/2019/4250613.
39. Wu, C.; Hwang, M. Risk stratification for lung adenocarcinoma on EGFR and TP53 mutation status, chemotherapy, and PD-L1 immunotherapy. *Cancer Med.* **2019**, *8*, 5850–5861, https://doi.org/10.1002/cam4.2492.
40. Jan, Y.-H.; Lai, T.-C.; Yang, C.-J.; Huang, M.-S.; Hsiao, M. A co-expressed gene status of adenylate kinase 1/4 reveals prognostic gene signature associated with prognosis and sensitivity to EGFR targeted therapy in lung adenocarcinoma. *Sci. Rep.* **2019**, *9*, 1–11, https://doi.org/10.1038/s41598-019-48243-9.
41. Wang, Z.; Wang, Z.; Niu, X.; Liu, J.; Wang, Z.; Chen, L.; Qin, B. Identification of seven-gene signature for prediction of lung squamous cell carcinoma. *OncoTargets Ther.* **2019**, *ume 12*, 5979–5988, https://doi.org/10.2147/ott.s198998.
42. Zhang, J.; Bing, Z.; Yan, P.; Tian, J.; Shi, X.; Wang, Y.; Yang, K. Identification of 17 mRNAs and a miRNA as an integrated prognostic signature for lung squamous cell carcinoma. *J. Gene Med.* **2019**, *21*, e3105, https://doi.org/10.1002/jgm.3105.
43. Liu, C.; Li, Y.; Wei, M.; Zhao, L.; Yu, Y.; Li, G. Identification of a novel glycolysis-related gene signature that can predict the survival of patients with lung adenocarcinoma. *Cell Cycle* **2019**, *18*, 568–579, https://doi.org/10.1080/15384101.2019.1578146.
44. Li, J.; Wang, H.; Li, Z.; Zhang, C.; Zhang, C.; Li, C.; Yu, H.; Wang, H. A 5-Gene Signature Is Closely Related to Tumor Immune Microenvironment and Predicts the Prognosis of Patients with Non-Small Cell Lung Cancer. *BioMed Res. Int.* **2020**, *2020*, 1–9, https://doi.org/10.1155/2020/2147397.

45. Liu, Y.; Guo, X.; Zhao, M.; Ao, H.; Leng, X.; Liu, M.; Wu, C.; Ma, J.; Zhu, J. Contributions and prognostic values of m 6 A RNA methylation regulators in non-small-cell lung cancer. *J. Cell. Physiol.* **2020**, *235*, 6043–6057, https://doi.org/10.1002/jcp.29531.
46. Liu, G.; Xie, W.; Jin, M.; Li, P.; Liu, L.; Liu, L.; Huang, G. Transcriptomic analysis reveals a WNT signaling pathway-based gene signature prognostic for non-small cell carcinoma. *Aging* **2020**, *12*, 19159–19172, https://doi.org/10.18632/aging.103724.
47. Li, Z.; Qi, F.; Li, F. Establishment of a Gene Signature to Predict Prognosis for Patients with Lung Adenocarcinoma. *Int. J. Mol. Sci.* **2020**, *21*, 8479, https://doi.org/10.3390/ijms21228479.
48. Liu, Y.; Wu, L.; Ao, H.; Zhao, M.; Leng, X.; Liu, M.; Ma, J.; Zhu, J. Prognostic implications of autophagy-associated gene signatures in non-small cell lung cancer. *Aging* **2019**, *11*, 11440–11462, https://doi.org/10.18632/aging.102544.
49. Li, J.; Li, Q.; Su, Z.; Sun, Q.; Zhao, Y.; Feng, T.; Jiang, J.; Zhang, F.; Ma, H. Lipid metabolism gene-wide profile and survival signature of lung adenocarcinoma. *Lipids Heal. Dis.* **2020**, *19*, 1–9, https://doi.org/10.1186/s12944-020-01390-9.
50. Li, J.; Li, X.; Zhang, C.; Zhang, C.; Wang, H. A signature of tumor immune microenvironment genes associated with the prognosis of non-small cell lung cancer. *Oncol. Rep.* **2020**, *43*, 795–806, https://doi.org/10.3892/or.2020.7464.
51. Fan, T.; Zhu, M.; Wang, L.; Liu, Y.; Tian, H.; Zheng, Y.; Tan, F.; Sun, N.; Li, C.; He, J. Immune profile of the tumor microenvironment and the identification of a four-gene signature for lung adenocarcinoma. *Aging* **2020**, *13*, 2397–2417, https://doi.org/10.18632/aging.202269.
52. Ma, C.; Luo, H.; Cao, J.; Zheng, X.; Zhang, J.; Zhang, Y.; Fu, Z. Identification of a Novel Tumor Microenvironment–Associated Eight-Gene Signature for Prognosis Prediction in Lung Adenocarcinoma. *Front. Mol. Biosci.* **2020**, *7*, https://doi.org/10.3389/fmolb.2020.571641.
53. Han, L.; Shi, H.; Luo, Y.; Sun, W.; Li, S.; Zhang, N.; Jiang, X.; Gong, Y.; Xie, C. Gene signature based on B cell predicts clinical outcome of radiotherapy and immunotherapy for patients with lung adenocarcinoma. *Cancer Med.* **2020**, *9*, 9581–9594, https://doi.org/10.1002/cam4.3561.
54. Ma, H.; Tong, L.; Zhang, Q.; Chang, W.; Li, F. Identification of 5 Gene Signatures in Survival Prediction for Patients with Lung Squamous Cell Carcinoma Based on Integrated Multiomics Data Analysis. *BioMed Res. Int.* **2020**, *2020*, 1–19, https://doi.org/10.1155/2020/6427483.
55. Bao, X.; Shi, R.; Zhao, T.; Wang, Y. Immune landscape and a novel immunotherapy-related gene signature associated with clinical outcome in early-stage lung adenocarcinoma. *J. Mol. Med.* **2020**, *98*, 805–818, https://doi.org/10.1007/s00109-020-01908-9.
56. Yu, X.; Zhang, X.; Zhang, Y. Identification of a 5-Gene Metabolic Signature for Predicting Prognosis Based on an Integrated Analysis of Tumor Microenvironment in Lung Adenocarcinoma. *J. Oncol.* **2020**, *2020*, 1–12, https://doi.org/10.1155/2020/5310793.
57. Tang, Y.; Jiang, Y.; Qing, C.; Wang, J.; Zeng, Z. Systematic construction and validation of an epithelialmesenchymal transition risk model to predict prognosis of lung adenocarcinoma. *Aging* **2020**, *13*, 794–812, https://doi.org/10.18632/aging.202186.
58. Jiang, H.; Xu, S.; Chen, C. A ten-gene signature-based risk assessment model predicts the prognosis of lung adenocarcinoma. *BMC Cancer* **2020**, *20*, 1–11, https://doi.org/10.1186/s12885-020-07235-z.
59. He, L.; Chen, J.; Xu, F.; Li, J. Prognostic Implication of a Metabolism-Associated Gene Signature in Lung Adenocarcinoma. *Mol. Ther. - Oncolytics* **2020**, *19*, 265–277, https://doi.org/10.1016/j.omto.2020.09.011.
60. Song, C.; Guo, Z.; Yu, D.; Wang, Y.; Wang, Q.; Dong, Z.; Hu, W. A Prognostic Nomogram Combining Immune-Related Gene Signature and Clinical Factors Predicts Survival in Patients With Lung Adenocarcinoma. *Front. Oncol.* **2020**, *10*, 1300, https://doi.org/10.3389/fonc.2020.01300.
61. Ma, X.; Ren, H.; Peng, R.; Li, Y.; Ming, L. Identification of key genes associated with progression and prognosis for lung squamous cell carcinoma. *PeerJ* **2020**, *8*, e9086, https://doi.org/10.7717/peerj.9086.
62. Zhao, Z.; He, B.; Cai, Q.; Zhang, P.; Peng, X.; Zhang, Y.; Xie, H.; Wang, X. A model of twenty-three metabolic-related genes predicting overall survival for lung adenocarcinoma. *PeerJ* **2020**, *8*, e10008, https://doi.org/10.7717/peerj.10008.
63. Sun, J.; Zhao, T.; Zhao, D.; Qi, X.; Bao, X.; Shi, R.; Su, C. Development and validation of a hypoxia-related gene signature to predict overall survival in early-stage lung adenocarcinoma patients. *Ther. Adv. Med Oncol.* **2020**, *12*, https://doi.org/10.1177/1758835920937904.
64. Wu, X.; Wang, L.; Feng, F.; Tian, S. Weighted gene expression profiles identify diagnostic and prognostic genes for lung adenocarcinoma and squamous cell carcinoma. *J. Int. Med Res.* **2019**, *48*, https://doi.org/10.1177/0300060519893837.
65. Li, W.; Li, X.; Gao, L.-N.; You, C.-G. Integrated Analysis of the Functions and Prognostic Values of RNA Binding Proteins in Lung Squamous Cell Carcinoma. *Front. Genet.* **2020**, *11*, https://doi.org/10.3389/fgene.2020.00185.
66. Guo, D.; Wang, M.; Shen, Z.; Zhu, J. A new immune signature for survival prediction and immune checkpoint molecules in lung adenocarcinoma. *J. Transl. Med.* **2020**, *18*, 1–14, https://doi.org/10.1186/s12967-020-02286-z.
67. Zhang, Y.; Zhang, X.; Lv, X.; Zhang, M.; Gao, X.; Liu, J.; Xu, Y.; Fang, Z.; Chen, W. Development and Validation of a Seven-Gene Signature for Predicting the Prognosis of Lung Adenocarcinoma. *BioMed Res. Int.* **2020**, *2020*, 1–10, https://doi.org/10.1155/2020/1836542.
68. Nema, R.; Shrivastava, A.; Kumar, A. Prognostic role of lipid phosphate phosphatases in non-smoker, lung adenocarcinoma patients. *Comput. Biol. Med.* **2020**, *129*, 104141, https://doi.org/10.1016/j.compbiomed.2020.104141.
69. Yang, X.; Wang, G.; Gu, R.; Xu, X.; Zhu, G. A signature of tumor DNA repair genes associated with the prognosis of surgically-resected lung adenocarcinoma. *PeerJ* **2020**, *8*, e10418, https://doi.org/10.7717/peerj.10418.
70. Wu, P.; Zheng, Y.; Wang, Y.; Wang, Y.; Liang, N. Development and validation of a robust immune-related prognostic signature in early-stage lung adenocarcinoma. *J. Transl. Med.* **2020**, *18*, 1–12, https://doi.org/10.1186/s12967-020-02545-z.

71. Wu, Q.; Wang, L.; Wei, H.; Li, B.; Yang, J.; Wang, Z.; Xu, J.; Zhou, Y.L.; Zhang, B. Integration of multiple key molecules in lung adenocarcinoma identifies prognostic and immunotherapeutic relevant gene signatures. *Int. Immunopharmacol.* **2020**, *83*, 106477, https://doi.org/10.1016/j.intimp.2020.106477.
72. Zou, X.; Hu, Z.; Huang, C.; Chang, J. A Seven-Gene Signature with Close Immune Correlation Was Identified for Survival Prediction of Lung Adenocarcinoma. *Med Sci. Monit.* **2020**, *26*, https://doi.org/10.12659/msm.924269.
73. Li, C.; Long, Q.; Zhang, D.; Li, J.; Zhang, X. Identification of a four-gene panel predicting overall survival for lung adenocarcinoma. *BMC Cancer* **2020**, *20*, 1–16, https://doi.org/10.1186/s12885-020-07657-9.
74. Zengin, T.; Önal-Süzek, T. Analysis of genomic and transcriptomic variations as prognostic signature for lung adenocarcinoma. *BMC Bioinform.* **2020**, *21*, 1–28, https://doi.org/10.1186/s12859-020-03691-3.
75. Zhao, M.; Li, M.; Chen, Z.; Bian, Y.; Zheng, Y.; Hu, Z.; Liang, J.; Huang, Y.; Yin, J.; Zhan, C.; et al. Identification of immune-related gene signature predicting survival in the tumor microenvironment of lung adenocarcinoma. *Immunogenetics* **2020**, *72*, 455–465, https://doi.org/10.1007/s00251-020-01189-z.
76. Cao, Y.; Lu, X.; Li, Y.; Fu, J.; Li, H.; Li, X.; Chang, Z.; Liu, S. Identification of a six-gene metabolic signature predicting overall survival for patients with lung adenocarcinoma. *PeerJ* **2020**, *8*, e10320, https://doi.org/10.7717/peerj.10320.
77. Zhao, J.; Guo, C.; Ma, Z.; Liu, H.; Yang, C.; Li, S. Identification of a novel gene expression signature associated with overall survival in patients with lung adenocarcinoma: A comprehensive analysis based on TCGA and GEO databases. *Lung Cancer* **2020**, *149*, 90–96, https://doi.org/10.1016/j.lungcan.2020.09.014.
78. Zheng, Z.; Deng, W.; Yang, J. Identification of 5-Gene Signature Improves Lung Adenocarcinoma Prognostic Stratification Based on Differential Expression Invasion Genes of Molecular Subtypes. *BioMed Res. Int.* **2020**, *2020*, 1–22, https://doi.org/10.1155/2020/8832739.
79. Ma, N.; Si, L.; Yang, M.; Li, M.; He, Z. A highly expressed mRNA signature for predicting survival in patients with stage I/II non-small-cell lung cancer after operation. *Sci. Rep.* **2021**, *11*, 1–18, https://doi.org/10.1038/s41598-021-85246-x.
80. Yan, D.; Chen, Y. Tumor mutation burden (TMB)-associated signature constructed to predict survival of lung squamous cell carcinoma patients. *Sci. Rep.* **2021**, *11*, 1–11, https://doi.org/10.1038/s41598-021-88694-7.
81. Ma, W.; Liang, J.; Liu, J.; Tian, D.; Chen, Z. Establishment and validation of an eight-gene metabolic–related prognostic signature model for lung adenocarcinoma. *Aging* **2021**, *13*, 8688–8705, https://doi.org/10.18632/aging.202681.
82. Zhang, X.; Shi, X.; Zhao, H.; Jia, X.; Yang, Y. Identification and Validation of a Tumor Microenvironment-Related Gene Signature for Prognostic Prediction in Advanced-Stage Non-Small-Cell Lung Cancer. *BioMed Res. Int.* **2021**, *2021*, 1–18, https://doi.org/10.1155/2021/8864436.
83. Yao, J.; Li, R.; Liu, X.; Zhou, X.; Li, J.; Liu, T.; Huo, C.; Qu, Y. Prognostic implication of glycolysis related gene signature in non-small cell lung cancer. *J. Cancer* **2021**, *12*, 885–898, https://doi.org/10.7150/jca.50274.
84. Yang, T.; Hao, L.; Cui, R.; Liu, H.; Chen, J.; An, J.; Qi, S.; Li, Z. Identification of an immune prognostic 11-gene signature for lung adenocarcinoma. *PeerJ* **2021**, *9*, e10749, https://doi.org/10.7717/peerj.10749.
85. Ma, Q.; Chen, Y.; Xiao, F.; Hao, Y.; Song, Z.; Zhang, J.; Okuda, K.; Um, S.-W.; Silva, M.; Shimada, Y.; et al. A signature of estimate-stromal-immune score-based genes associated with the prognosis of lung adenocarcinoma. *Transl. Lung Cancer Res.* **2021**, *10*, 1484–1500, https://doi.org/10.21037/tlcr-21-223.
86. Wang, S.; Wu, C.; Ma, D.; Hu, Q. Identification of a ferroptosis-related gene signature (FRGS) for predicting clinical outcome in lung adenocarcinoma. *PeerJ* **2021**, *9*, e11233, https://doi.org/10.7717/peerj.11233.
87. Xu, Z.; Zhang, S.; Nian, F.; Xu, S. Identification of a glycolysis-related gene signature associated with clinical outcome for patients with lung squamous cell carcinoma. *Cancer Med.* **2021**, *10*, 4017–4029, https://doi.org/10.1002/cam4.3945.
88. Tu, Z.; He, X.; Zeng, L.; Meng, D.; Zhuang, R.; Zhao, J.; Dai, W. Exploration of Prognostic Biomarkers for Lung Adenocarcinoma Through Bioinformatics Analysis. *Front. Genet.* **2021**, *12*, https://doi.org/10.3389/fgene.2021.647521.
89. Zhu, G.; Huang, H.; Xu, S.; Shi, R.; Gao, Z.; Lei, X.; Zhu, S.; Zhou, N.; Zu, L.; De Mello, R.A.; et al. Prognostic value of ferroptosis-related genes in patients with lung adenocarcinoma. *Thorac. Cancer* **2021**, *12*, 1890–1899, https://doi.org/10.1111/1759-7714.13998.
90. Xu, R.; Lu, T.; Wang, J.; Zhang, L. A novel protein ubiquitination-related five-gene signature predicts overall survival in patients with lung adenocarcinoma. *Aging* **2021**, *13*, 8510–8523, https://doi.org/10.18632/aging.202663.
91. Chen, J.; Zhou, R. Tumor microenvironment related novel signature predict lung adenocarcinoma survival. *PeerJ* **2021**, *9*, e10628, https://doi.org/10.7717/peerj.10628.
92. Yang, L.; written on behalf of the AME Thoracic Surgery Collaborative Group; Zhang, R.; Guo, G.; Wang, G.; Wen, Y.; Lin, Y.; Zhang, X.; Yu, X.; Huang, Z.; et al. Development and validation of a prediction model for lung adenocarcinoma based on RNA-binding protein. *Ann. Transl. Med.* **2021**, *9*, 474–474, https://doi.org/10.21037/atm-21-452.
93. Miao, T.-W.; Du, L.-Y.; Xiao, W.; Mao, B.; Wang, Y.; Fu, J.-J. Identification of Survival-Associated Gene Signature in Lung Cancer Coexisting With COPD. *Front. Oncol.* **2021**, *11*, https://doi.org/10.3389/fonc.2021.600243.
94. Wang, Z.; Embaye, K.S.; Yang, Q.; Qin, L.; Zhang, C.; Liu, L.; Zhan, X.; Zhang, F.; Wang, X.; Qin, S. Establishment and validation of a prognostic signature for lung adenocarcinoma based on metabolism-related genes. *Cancer Cell Int.* **2021**, *21*, 1–16, https://doi.org/10.1186/s12935-021-01915-x.
